# Early and Late Effects of Semantic Distractors on Electroencephalographic Responses During Overt Picture Naming

**DOI:** 10.3389/fpsyg.2019.00696

**Published:** 2019-03-28

**Authors:** Andrea Krott, Maria Teresa Medaglia, Camillo Porcaro

**Affiliations:** ^1^Centre for Human Brain Health, School of Psychology, University of Birmingham, Birmingham, United Kingdom; ^2^Institute of Cognitive Sciences and Technologies (ISTC) – National Research Council (CNR), Rome, Italy; ^3^S. Anna Institute and Research in Advanced Neurorehabilitation (RAN), Crotone, Italy; ^4^Department of Information Engineering, Università Politecnica delle Marche, Ancona, Italy

**Keywords:** electroencephalography (EEG), oscillations, word production, picture-word interference paradigm, semantic competition, lexical competition, response exclusion, overt picture naming

## Abstract

This study investigated the nature of the interference effect of semantically related distractors in the picture-word interference paradigm, which has been claimed to be caused by either competition between lexical representations of target and distractor or by a late response exclusion mechanism that removes the distractor from a response buffer. EEG was recorded while participants overtly named pictures accompanied by categorically related versus unrelated written distractor words. In contrast to previous studies, stimuli were presented for only 250 ms to avoid any re-processing. ERP effects of relatedness were found around 290, 470, 540, and 660 ms post stimulus onset. In addition, related distractors led to an increase in midfrontal theta power, especially from about 440 to 540 ms, as well as to decreased high beta power between 40 and 110 ms and increased high beta power between 275 and 340 ms post stimulus onset. Response-locked analyses showed no differences in ERPs, however increased low and high beta power for related distractors in various time windows, most importantly a high beta power increase between −175 and −155 ms before speech onset. These results suggest that the semantic distractor effect is a combination of various effects and that the lexical competition account and the response exclusion account each capture a part, but not all aspects of the effect.

## Introduction

One of the core processes of language production is the selection of the appropriate representations for a particular concept from our mental lexicons. Most models of speech production agree that word production involves a number of processing stages, namely at a conceptual level, a lexical level, a morphophonological level and a phonetic/articulatory level (e.g., Dell, 1986; Levelt et al., 1999). Models also generally agree that during the lexical access of a target word (e.g., *banana*) not only its lexical representation is activated, but that of semantically related words (e.g., *apple, orange, pear* etc.) (Dell, 1986; Caramazza, 1997; Levelt et al., 1999; Goldrick and Rapp, 2002; Indefrey, 2011). However, models do disagree on whether the co-activated lexical representations compete for selection or not. Some models do assume lexical selection by competition (e.g., Schriefers et al., 1990; Roelofs, 1992; Starreveld and La Heij, 1996; Levelt et al., 1999; Bloem and La Heij, 2003; Abdel Rahman and Melinger, 2009). More precisely, they assume that the co-activation of semantically related representations interferes with the production of the target word, slowing down the production process and leading to slower word production. This is especially the case if a competitor is highly activated. The higher the activation of the competitor, the slower the production of the target. The exact mechanism causing the slowing varies between models. For instance, Cutting and Ferreira (1999) proposed the existence of lateral inhibitory links between lexical representations, and the WEAVER++ model (Roelofs, 1992) proposes that a word is selected for production only once its activation exceeds those of other representations by a critical amount. In contrast, other models (e.g., Dell, 1986; Caramazza, 1997) assume that the co-activation of other lexical items does not affect the selection of the target word. Instead, only the target’s own activation level affects the speed of its naming process. However, a co-activated word might be selected for production if its activation is higher than that of the target. This would lead to a speech error.

The assumption of lexical selection by competition was originally supported by findings of a particular experimental paradigm, namely the picture-word interference (PWI) paradigm. In this paradigm, participants are asked to name pictures with visually or auditorily superimposed distractor words. Numerous studies have found that distractors from the same semantic category as the target (e.g., ‘cat’ as a distractor to the target ‘fish’) slow down naming times when compared to unrelated distractors (e.g., ‘cup’) (e.g., Lupker, 1979; Glaser and Düngelhoff, 1984; Schriefers et al., 1990). This finding has been called the “semantic interference effect.” The lexical selection by competition interpretation of the semantic interference effect states that the representation of an unrelated distractor receives activation from the distractor word only, while a semantically related distractor receives activation from both the distractor word and the target due to spreading activation at the conceptual level. This is assumed to result in higher activation of related than unrelated distractors and as a consequence to stronger competition with the target. This in turn slows down response times. Importantly, the competition between target and related words is assumed to take place at lexical level.

This traditional lexical competition account of the semantic interference effect has been questioned. The alternative account that has been proposed is the so-called “response exclusion account.” This account extends models that do not assume lexical competition (e.g., Caramazza, 1997). It proposes that a semantic distractor affects the naming of a picture at two stages: during early semantic processing of the picture and a late processing stage, i.e., when a fully planned response to the distractor needs to be removed from a response buffer (Finkbeiner and Caramazza, 2006; Mahon et al., 2007; Janssen et al., 2008; Dhooge and Hartsuiker, 2010, 2012). The early semantic effect is a semantic priming effect and thus facilitatory. The semantic interference effect and thus the slowing down is caused by the need to remove the distractor from the response buffer. This buffer is occupied by the response to the written distractor because it is processed faster than the picture. The removal is assumed to be affected by general semantic constraints or “response-relevant criteria.” It is checked whether the word in the buffer meets these constraints in order to be an acceptable response. Because unrelated distractors are less response relevant, their removal is easier and faster than the removal of a related distractor. This leads to slower responses for related than unrelated distractors.

Evidence for the accounts has primarily been found by manipulating the type of the distractor of the PWI paradigm (e.g., Alario et al., 2000; Miozzo and Caramazza, 2003; Abdel Rahman and Melinger, 2007; Dhooge and Hartsuiker, 2010; Roelofs et al., 2011). But both lexical competition accounts and the response exclusion account are able to explain the behavioral findings that have been reported to date (Roelofs et al., 2011). Deeper insights might therefore come from neuroimaging studies or studies using electroencephalography (EEG).

Studies using functional magnetic resonance imaging (fMRI) have suggested that the two accounts are not necessarily mutual exclusive explanations of the semantic interference effect (de Zubicaray et al., 2012a,b). For instance, de Zubicaray et al. (2012b) manipulated frequency and age-of-acquisition of distractors in a PWI paradigm. The age-of-acquisition effect was associated with the left middle and posterior middle temporal gyrus (MTG), areas generally thought to play a role in lexical semantic processing (e.g., Indefrey and Levelt, 2004; Acheson et al., 2011; Indefrey, 2011). This finding is therefore in accordance with the assumption of the lexical competition account that distractors compete with the target at a lexical level. In contrast, manipulating distractor frequency affected neural activity in posterior superior temporal cortices (pSTG) and left premotor cortices. The pSTG has been related to the retrieval of phonological word forms / phonological encoding, and premotor cortical areas have been related to articulation (e.g., Indefrey and Levelt, 2004; Wilson et al., 2009; Acheson et al., 2011; Indefrey, 2011). Because these processes are assumed to occur close to response, the involvement of these areas is in accordance with the assumption of the response exclusion account of a late removal of a fully planned distractor from a response buffer.

Because the two opposing accounts of the PWI effect make different predictions concerning the timing of the effect, EEG with its strong temporal resolution seems to be particularly suited to distinguish between the accounts. While the lexical competition account predicts an effect during the time of lexical selection processes, the response exclusion account predicts a late effect close to speech onset due to the exclusion of the distractor from the output buffer. A number of studies have studied ERPs and oscillatory EEG activity to trace the time course of the PWI effect (e.g., Greenham and Stelmack, 2001; Hirschfeld et al., 2008; Dell’Acqua et al., 2010; Aristei et al., 2011; Hoshino and Thierry, 2011; Blackford et al., 2012; Piai et al., 2012; Zhu et al., 2015; Shitova et al., 2016, 2017; Wong et al., 2017; Rose et al., 2018). The dominant ERP pattern of results seems to suggest a modulation of the N400 component (Dell’Acqua et al., 2010; Blackford et al., 2012; Wong et al., 2017; Zhu et al., 2015). Unrelated distractors (e.g., picture of a banana with the superimposed distractor ‘*house*’) compared to related category distractors (distractor ‘*apple*’) have been found to show a more negative N400 component roughly between 250 and 450 ms, an N400-like effect (see also Shitova et al., 2016, 2017 for identity distractors, e.g., distractor ‘banana’). Given the timing of these effects, they have been interpreted as being related to lexical-semantic access and therefore in line with the lexical selection by competition hypothesis (but see Blackford et al., 2012).

While a N400 modulation is a common result, ERP differences between semantically related and unrelated distractors have not always been found (Greenham and Stelmack, 2001; Hirschfeld et al., 2008; Aristei et al., 2011; Piai et al., 2012; Shitova et al., 2016) and the nature of the ERP effects that have been reported varies (Dell’Acqua et al., 2010; Aristei et al., 2011; Hoshino and Thierry, 2011; Zhu et al., 2015; Wong et al., 2017; Rose et al., 2018). Hoshino and Thierry (2011) reported a much shorter effect at 350–400 ms, in addition to an earlier effect during 200–260 ms (reduced negative deflection for related distractors compared to unrelated distractors). However, their participants were Spanish-English bilinguals, meaning that their effects might partly reflect interference and/or inhibition of their other language. Dell’Acqua et al. (2010) reported an additional negativity between 50 and 200 ms, which they interpreted as being due to feedback processes from the semantics of the picture to the processing of the distractor word. While the studies presented so far all found reduced negative deflections for semantically related compared to unrelated distractors, Aristei et al. (2011) found the opposite effect between 200 and 400 ms. But their paradigm was more complex, as they presented stimuli in semantically related and unrelated blocks. This is also the only study that found effects when presenting distractors auditorily instead of visually. Rose et al. (2018) reported an anterior negativity for related distractors roughly between 340 and 450 ms. Furthermore, studies varied with respect to the distribution of the effects. While some found wide-spread central(-parietal) effects that resemble N400 effects (Dell’Acqua et al., 2010; Hoshino and Thierry, 2011; Zhu et al., 2015; Rose et al., 2018), others found (additional) anterior effects (Aristei et al., 2011; Blackford et al., 2012; Wong et al., 2017; Rose et al., 2018).

Leaving the differences between the findings of previous ERP studies using the PWI paradigm aside, in almost all studies, stimuli (i.e., pictures and distractors) were visually presented and remained on the screen until participants responded (but see Aristei et al., 2011; Blackford et al., 2012). This opens up the possibility that pictures and words are processed again, for instance, during later monitoring processes. This in turn means that ERP effects might partly be affected by such re-processing. We decided to avoid any effects of re-processing by adapting a variant of the paradigm where the stimuli are presented on the screen for a much shorter time (e.g., Schriefers et al., 1990; Zwitserlood et al., 2000), namely for 250 ms only. This also means that any potential late effects that we would find cannot be due to the re-processing of the stimuli.

Furthermore, almost all ERP effects for related versus unrelated distractors reported to date ended before 400/450 ms (550 ms in case of Blackford et al., 2012) and are, at least in terms of timing, in line with the “selection by competition” hypothesis [but see Blackford et al. (2012) interpretation]. In contrast to ERP findings so far, the response exclusion account predicts an effect close to speech onset. To our knowledge, only one PWI study to date has investigated such late ERP responses (Wong et al., 2017), most likely due to a fear of “distortion” of the EEG during this time window by speech motor artifacts (but see Piai et al., 2014, for a response-locked time-frequency analysis in a related paradigm). Wong et al. (2017) investigated late effects by a response-locked analysis. They found an ERP effect only when comparing associative distractors (semantically but not categorically related to the picture) with unrelated distractors, not for categorical distractors, as of interest here. Since the response exclusion account makes predictions about processes very close to responses, it is important to study these later processes with the right approach, i.e., ideally with a response-locked analysis.

There are relatively few studies that have investigated oscillatory EEG activity during a PWI study (Piai et al., 2012; Shitova et al., 2017). Piai et al. (2012) reported a central power increase in the beta band (12–30 Hz) for categorically related relative to unrelated distractors between 230 and 370 ms. Shitova et al. (2017) reported a midfrontal theta power increase (4–6 Hz) for semantically related versus identical distractors from about 200 ms post stimulus onset until end of the trial (see also theta activity increase in the MEG study by Piai et al., 2014). The beta activity increase for categorically related versus unrelated distractors in Piai et al. (2012) study fits the selection by competition assumption due to its timing. The long-lasting theta power increase from ∼200 ms by Shitova et al. (2017), on the other hand, suggests ongoing heightened cognitive control from about 200 ms onward (e.g., Cavanagh and Frank, 2014) and might therefore mean that neither the lexical selection account nor the response exclusion account capture all aspects of the PWI effect. It seems that a ‘conflict’ between the picture and the distractor is identified early during speech preparation, leading to this heightened control. The maintained increased control right up to speech onset suggests that enhanced cognitive control might be required during various stages of speech preparation, including lexical selection, but also during later processing stages. Taken together, investigating oscillatory EEG activity can provide additional information about the timing of processes in the PWI paradigm and therefore potential evidence for either or both of the opposing accounts of the PWI effect.

In sum, since leaving stimuli in the PWI paradigm on the screen until response could lead to re-processing, we presented stimuli for only 250 ms. In addition, previous EEG studies have almost exclusively studied ERPs locked to the stimulus and have focused on ‘early’ effects. While this approach can provide evidence for the lexical competition account, it is limited in its ability to reveal evidence for the response exclusion account, which is expected to occur close to speech onset due to the exclusion of the distractor from the output buffer. In order to minimize any contamination of speech motor artifacts, we used a muscle artifact attenuation procedure, namely Speech Artifact Removal by Independent Component Analysis (SAR-ICA) (Porcaro et al., 2015). Furthermore, we investigated effects for both ERPs and oscillatory activity in order to widen the search space. In line with the predictions of the two accounts of the PWI effect, we expected to see effects during the time window of lexical processing and/or after the preparation of a phonological word, thus close to speech onset.

## Materials and Methods

### Participants

Eighteen participants (mean age 23.3, SD 3.7, 10 males) took part in the experiment and received either course credit or £20 for their participation. All were right-handed determined by the Edinburgh handedness inventory (Oldfield, 1971). All had normal or corrected-to-normal vision and were monolingual native English speakers. Ethical approval for the research was obtained from the Ethics Board of the School of Psychology at Birmingham University.

### Materials

Twenty-four line-drawings of common objects were selected from the Snodgrass and Vanderwart (1980) picture set. They were paired with semantically related distractor words taken from the same semantic category as the target [e.g., banana (target picture) – orange (distractor word)]. For category membership, we followed Abdel Rahman and Melinger (2007) definition as items that share a semantic category node (e.g., fruit) and specific features (e.g., grows on trees, is sweet etc). We chose from a wide range of categories. Unrelated target-distractor pairs were created by re-pairing targets and distractor words (see Appendix A for a complete list of material). This ensured that related and unrelated distractors as a group were perfectly matched for all possible variables (length, frequency etc.).

### Procedure

**FIGURE 1:**
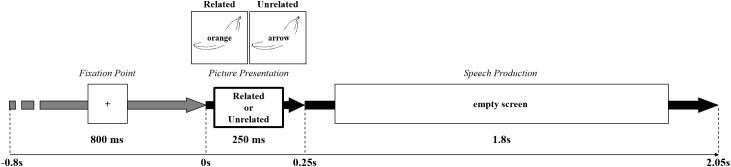
Experimental Procedure. The beginning of the trial was signaled by a “+” appearing in the center of the screen for 800 ms, followed by the picture presentation with a related or unrelated distractor for 250 ms. During speech production, an empty screen appeared for minimum 1800 ms. The Figure shows an example of a semantically related and unrelated distractor.

Participants were seated in a quiet and normally illuminated test room 1 m away from a 17″ monitor with a resolution of 800 × 600 pixels. They first familiarized themselves with the pictures and their names by studying a picture booklet. In the experiment, participants saw the pictures with superimposed written distractor words as shown in Figure 1. They were instructed to name the pictures, while ignoring the distractor words, and to respond as fast and as accurately as possible. E-Prime (Psychological Software Tools, Inc.) was used to control stimulus presentation and data collection. Responses were recorded for off-line error analysis and response times were measured using a voicekey (PST SRBox). Each trial began with the presentation of a fixation cross for 800 ms, followed by a stimulus appearing for 250 ms and a subsequent empty screen. This prevented participants from re-reading the distractor word during later processing stages (e.g., during monitoring) and also minimized eye movements. The maximum response time was 2200 ms and each trial lasted up to 3000 ms. Participants were instructed to keep movements other than speaking to a minimum and to try to blink only after providing a response.

Two stimulus lists were created, each featuring all objects, combined with one of the distractors. Half of the objects in each list were combined with the related distractor, and the other half with the unrelated distractor. The order of the items within the lists was random. In order to increase the number of trials for the analysis, each participant saw each list eight times in alternation, leading to 192 trials per condition (total 384 trails). The order of the lists was counterbalanced across participants. There was a short pause after the completion of every second list. The experiment lasted for about 75 min (including breaks).

### EEG and EMG Recording Parameters

Electroencephalograms (EEG) were acquired using a 128 channel BioSemi Active Two EEG system, with electrodes placed in a nylon cap according to the 10–5 system (Oostenveld and Praamstra, 2001). Horizontal and vertical electrooculograms and upper and lower lip electromyograms (EMG) were monitored by bipolar derivations. The data were sampled at 512 Hz. They were off-line referenced to an average of the left and right mastoids, baseline-corrected using the average EEG activity in the interval between −100 ms and stimulus presentation and filtered with a band-pass of 0.1–30 Hz using a finite impulse response (FIR) filter.

## Data Analysis

For statistical analyses of both the behavioral and ERP data we excluded all trials with missed or incorrect responses, with disfluencies and self-repairs. We also considered responses with reaction times below 250 ms as voicekey errors and responses above 1800 ms as outliers and removed those from the analyses (<0.5% of the data). For the behavioral data, we compared both speech onset times and number of errors (missed and incorrect responses, disfluencies and self-repairs) for pictures with related and unrelated distractors.

### SAR-ICA Analysis

The data were cleaned from motor artifacts using the previously developed Speech Artifact Removal by Independent Component Analysis (SAR-ICA) procedure (Porcaro et al., 2015), a ICA procedure similar to that used for the attenuation of other artifacts (Barbati et al., 2004; Makeig et al., 2004, Medaglia et al., 2009; Porcaro et al., 2006, 2009, 2011). The SAR-ICA procedure decomposes the signal into independent components (ICs) on the basis of statistical properties of the signal. Using information from averaged trials, single trials, topographical distributions, localizations, and correlations with the lip EMG, ICs are classified into the following clusters: cleaned data, articulatory speech artifacts, environmental noise, and ocular artifacts. The data at scalp electrodes for the cleaned data cluster are obtained by retro-projecting the selected independent components. We have presented the SAR-ICA for the present dataset in detail in Porcaro et al. (2015), together with a validation of our method and a comparison with other methods for motor artifact removal previously reported in the literature. We found a high validity and superiority of our method over previous other approaches. Therefore, cleaning the data from speech motor artifacts using the SAR-ICA method should provide us with overall reliable data, i.e., for both early time-windows and those close to speech onset.

### ICA on ERP Data

In order to rule out that we are dealing with latency effects instead of amplitude differences for the ERP peaks between 440 and 670 ms, we conducted ICA analyses on the ERPs of related and unrelated distractor conditions.

### Cluster-Based Permutation Tests

To identify effects of distractor relatedness on brain responses, we examined ERP responses to related versus unrelated distractors with both a stimulus-locked and a response-locked analysis. However, we only found significant differences between conditions in the stimulus-locked analysis. We therefore report only those. For the stimulus-locked analysis, we investigated a window that roughly covered processes before speech onset, i.e., 0–800 ms. We split the time window into two equal-sized windows, 0–399 ms for early effects and 400–800 ms for late effects. Because ERP differences that had previously been reported for semantic distractors in PWI studies occurred in various time-windows and because ERP differences between categorically related and unrelated distractors closer to responses had previously either not been studied or not been found, we investigated ERP effects by taking an explorative approach. For that we submitted ERPs to repeated measures, two-tailed cluster mass permutation tests with a family-wise alpha level of 0.01, using the Mass Univariate ERP Toolbox^1^ (Bullmore et al., 1999; Groppe et al., 2011). We included all time points between 0 and 399 ms and 400 and 800 ms at all 128 scalp electrodes in the test and used 5.44 cm (default setting of the toolbox) to determine electrodes as spatial neighbors. Repeated measures *t*-tests were performed for each comparison using the original data and 2500 random within-participant permutations of the data. For each permutation, all t-scores corresponding to uncorrected *p*-values of 0.01 or less were formed into clusters. The sum of the t-scores in each cluster is the “mass” of that cluster and the most extreme cluster mass in each of the 2501 sets of tests was recorded and used to estimate the distribution of the null hypothesis.

### Time Frequency Analysis

Stimulus and response-locked time-frequency event related synchronization and desynchronization (ERS/ERD) analyses were performed using a Morlet wavelet, with a constant parameter equal to seven which offered the best compromise between time and frequency resolution, for both related and unrelated conditions and for each subject. Statistical significance of power changes compared to a 500 ms pre-stimulus baseline for stimulus-locked and from −1500 to −1000 ms respect to the speech onset baseline for the response-locked were evaluated with a resampling bootstrap technique and a threshold of *p* = 0.05. Non-significant changes were set to zero (Barbati et al., 2008; Porcaro et al., 2017). Given previous findings of theta and beta band changes at mid-frontal areas, especially those for very similar paradigms as the present one (Piai et al., 2012; Shitova et al., 2017), we investigated such time frequency changes at FCz, integrating the time frequency analysis as described above in theta (4–8 Hz), low beta (14–21 Hz) and high beta band (22–30 Hz). Once the theta, low and high beta band dynamics for each subject were obtained for both related and unrelated conditions, a pointwise statistical analysis was performed on the three waveforms conducting two-sample permutation *t*-tests (10,000 permutations) (Porcaro et al., 2019).

## Results

Effects of distractor relatedness on speech onset times were in line with previous findings. Average speech onset times were faster for unrelated (844 ms, SD 206) than related distractors (865 ms, SD 217) [*t*(17) = 4.3, *p* < 0.001]. The average error rates were small and did not significantly differ between related and unrelated distractors (semantically related: 1.4%; unrelated 1.1%).

In order to rule out that the repetition of the stimuli had an effect on response times, as found for instance by La Heij and Van den Hof (1995), we conducted an ANOVA with Relatedness (related, unrelated) and Block (Block 1 to Block 8) as independent variables and RTs as dependent variables. While we still found an effect of Relatedness, we did not find a significant effect of Block or a significant Block x Relatedness interaction (both *p*s > 0.05). Thus, repeating stimuli did not affect the relatedness effect in the behavioural results in our experiment.

**FIGURE 2:**
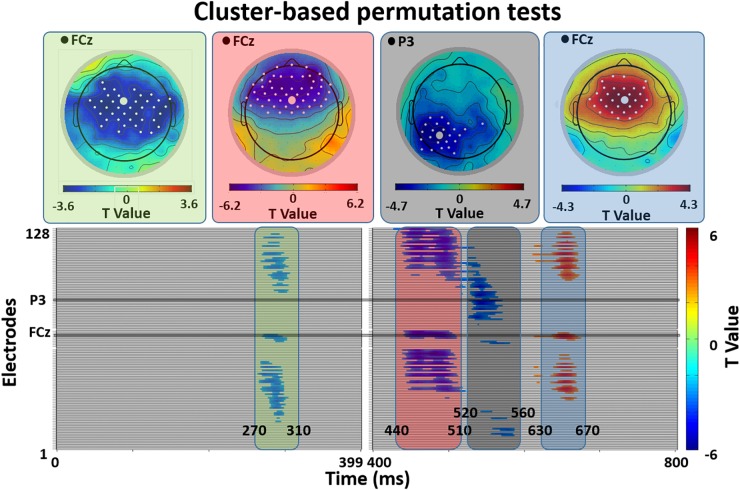
Cluster-based permutation tests of ERP differences between Semantically Related and Unrelated distractor conditions. The lower panels plot significance of ERP differences for all 128 electrodes in four different time-windows post stimulus onset (270–310 ms, 440–510 ms, 520–560 ms, 630–670 ms). The upper panels show the scalp distribution of these effects (related vs. unrelated distractors). White dots indicate electrodes with significant differences. Large white dots indicate the illustrative electrodes in Figure 3.

**FIGURE 3:**
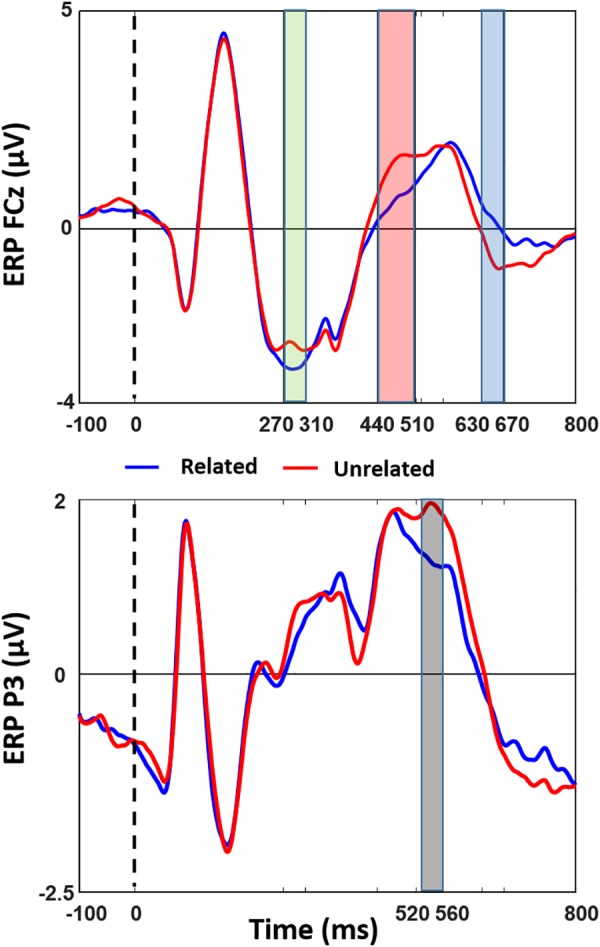
ERP comparison of Semantically Related and Unrelated Distractor conditions. Averaged stimulus-locked ERPs of related and unrelated distractor conditions are shown at illustrative electrodes (FCz and P3). Colors of effects are kept the same as in Figure 2.

Figure 2 shows the results of the cluster-based permutation test of ERP differences on all 128 electrodes between the related and unrelated distractor conditions, and Figure 3 shows averaged waveforms for electrodes FCz and P3 illustrating all effects. We found prominent ERP differences around four peaks: 290 ms (270–310 ms), 470 ms (440–510 ms), 540 ms (520–560 ms), and 660 ms (630–670 ms). The window around the 290 ms peak showed a larger negativity for related than unrelated distractors, widely distributed over the electrode space (see Figure 2, first topography, little white dots represent significant electrodes and big white dots represent the topographic position of the FCz electrode). For the 470 ms peak window we found a reduced positivity for related compared to unrelated distractors at anterior electrode sites (see Figure 2, second topography). The direction of the effect was the same at the 540 ms peak, but located at posterior left sides (see Figure 2, third topography). The 660 ms peak window showed a reduced negativity for related than unrelated distractors at anterior-central sites (see Figure 2, fourth topography)^2^.

Similar to the behavioral analysis, we also investigated whether the repetition of stimuli had an effect on the ERP effects. Since a single block only consisted of 24 trials per condition, which is too few for reliable significant condition differences, we conducted the same cluster-based permutation tests as for the total data on the first and final (i.e., fourth) quartile of the data. For both subsets of trials, we found very similar effects and in the same time windows as for all trials combined. In addition, there were no significant ERP differences between the first and fourth quartile, for neither the related nor the unrelated condition (see Supplementary Figure S1). Furthermore, we checked whether the later three ERP effects (those between 440 ms and 670 ms) might be due to differences in latencies instead of amplitudes. However, we found that latencies of these effects were not different between related and unrelated distractor conditions [peak around 470 ms: *t*(17) = 1.06, *p* = 0.302; peak around 540 ms: *t*(17) = 1.04, *p* = 0.314; peak around 660 ms: *t*(17) = 0.39, *p* = 0.701]. We are therefore indeed dealing with amplitude differences. Furthermore, the waveforms in Figure 3 suggest that related and unrelated conditions might have led to different ERP components. However, this was not the case. Performing ICA analyses on the ERPs of the related and unrelated distractor conditions showed three different components that created the three late peaks for both related and unrelated distractor conditions. This means that the three late peaks were independent from each other (see Supplementary Figure S2), meaning we have no evidence for different underlying mechanisms in the two conditions.

**FIGURE 4:**
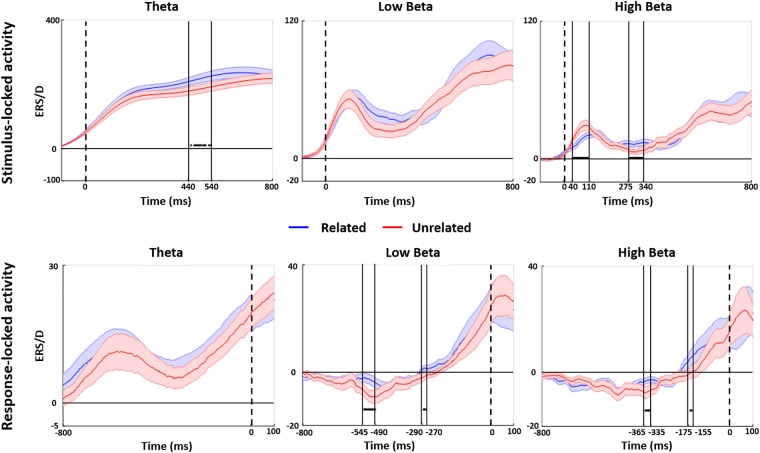
Stimulus and response-locked time-frequency analyses. Point-by-point stimulus-locked (upper panel) and response-locked (lower panel) analyses of related (blue solid line) and unrelated (red solid line) distractors are shown for the FCz electrode with the shaded area of the same color highlighting standard error. Horizontal black thick lines indicate a significant group difference between related vs. unrelated distractor conditions (permutation *t*-test at *p* = 0.05).

Figure 4 shows the results of the time-frequency analysis at FCz. The stimulus-locked analysis showed increased theta power for related versus unrelated distractors from 440 to 540 ms post stimulus. We also found a decrease in high beta band power for related versus unrelated distractors between 40 and 110 ms and an increase in high beta band power from 275 to 340 ms post stimulus onset. The response-locked analysis at FCz showed various increases in low and high beta band power in the final ∼550 ms before speech onset, with the latest increase from −175 to −155 ms before speech onset (other effects: low beta: −545 to −490 ms and −290 to −270 ms pre-speech onset; high beta: −275 to −340 ms post stimulus onset; −365 to −335 ms pre speech onset). There were no differences in any other frequency bands.

## Discussion

The aim of the present study was to investigate the nature of the semantic interference effect in the PWI paradigm with regard to the lexical competition account and the response exclusion account by focusing on EEG evidence. We conducted a PWI experiment in which participants named pictures superimposed with semantically related or unrelated written distractor words. Speech onset latencies, error rates, ERPs, and changes in oscillatory activity were investigated. As explained in the introduction, we aimed to avoid any potential re-processing of the stimuli and therefore presented them for a short period of time. In addition, existing EEG studies of PWI had typically considered ERP correlates until about 400/500 ms after picture onset. Similar to Wong et al. (2017), we explored also whether later ERPs as well as oscillatory activity, i.e., those closer to speech onset, were systematically affected by the relationship between targets and distractors. This enabled us to test the prediction of the response exclusion account to find effects close to speech onset caused by the assumed removal of fully planned responses form a response buffer.

In terms of behavioral results we replicated previous findings (e.g., Schriefers et al., 1990). Participants named the pictures more slowly when the distractors were semantically related than when they were unrelated to the targets. In terms of ERPs, only the stimulus-locked analysis showed effects of distractor relatedness. We found several effects of semantic relatedness (i.e., for related relative to unrelated distractors) from around 270 ms to about 670 ms post stimulus onset: A widespread negativity around 290 ms, a reduced anterior positivity around 470 ms, a reduced left-posterior positivity around 540 ms, and a reduced anterior-central negativity around 660 ms. In terms of oscillatory activity, we found that related distractors led to an increase in midfrontal theta power from about 450 to 540 ms, as well as to decreased high beta power between 40 and 110 ms and increased beta power between 275 and 340 ms, all post stimulus onset. A response-locked analysis showed that related distractors led to increased high and low beta power at various times before response onset, most importantly a high beta power increase from about −175 to −155 ms before speech onset.

In order to decide whether these effects are evidence for the lexical selection account and/or the response exclusion account, one needs estimates of when the accounts predict effects to occur. The lexical competition account assumes effects to occur during lemma retrieval. The response exclusion account is unfortunately not very specific in terms of when exactly the exclusion of the response from the articulatory buffer occurs. But, if fully prepared phonological responses are excluded (Finkbeiner and Caramazza, 2006; Mahon et al., 2007; Janssen et al., 2008; Dhooge and Hartsuiker, 2010, 2012), then effects should be seen from the point when a fully prepared phonological word is available.

Indefrey and Levelt (2004) and Indefrey (2011) meta-analyses, while somewhat limited as they are based on Levelt et al. (1999) model of word production, provide timing estimates for the different stages of speech production for a 600 ms response. According to these estimates, lemma retrieval should start at about 200 ms after picture presentation, phonological code retrieval at about 275 ms, self-monitoring at around 355 ms and phonetic encoding at about 455 ms. However, since these estimates are based on a response of 600 ms, they have to be rescaled for our longer response time of ∼850 ms. There are two ways of rescaling these estimates, proportional and informed rescaling (Indefrey, 2011; Roelofs and Shitova, 2017). Proportional rescaling assumes that all stages of speech production take longer and therefore all stages contribute to the prolonging of the responses and with equal weight. In contrast, informed rescaling means that only those stages are lengthened that are assumed to take longer in the given paradigm. Since the two approaches lead to very similar estimates for our study, we will focus here on the informed rescaling method. A picture naming study by Bürki (2017), which compared ERPs to pictures with unrelated written distractor words to those without distractors, provides some information for informed rescaling, at least for the first 250 ms post stimulus onset, that is until the stimuli disappeared in our experiment. Bürki (2017) found differences in ERP amplitudes emerged from about 200 ms post stimulus presentation. We therefore did not rescale the time window of conceptual preparation (initial 200 ms). In addition, we assumed that phonetic encoding would not take longer than in faster responses (final 145 ms), given that at this point the speaker has decided which word to produce (but note that rescaling this time window as well does not change the interpretations of our effects described below). Distributing the additional time (250 ms) that participants in our study took compared to the benchmark time of 600 ms evenly over the time windows of lemma retrieval and phonological retrieval/syllabification/monitoring would mean that lemma retrieval would last from about 200 to 400 ms, phonological code retrieval and syllabification from about 400 to 705 ms, followed by phonetic encoding. In addition, self-monitoring should start sometime between 500 and 605 ms.

Based on these estimates, the lexical competition account predicts effects in our study around 200 to 400 ms. The response exclusion account predicts effects right before ∼700 ms post stimulus presentation or right before ∼150 ms pre-response. If Dhooge and Hartsuiker (2010, 2012) are correct in that the speech monitor is involved in the response exclusion, then effects might also occur from about 500 to 605 ms. But note that this monitor would not work on fully prepared phonological responses as usually assumed in the response exclusion account (Finkbeiner and Caramazza, 2006; Mahon et al., 2007; Janssen et al., 2008; Dhooge and Hartsuiker, 2010, 2012).

We can now compare our findings with these estimates. Our earliest ERP effect (270 – 310 ms) falls into the lemma retrieval time window and could therefore be related to increased lexical competition caused by the relatedness of the pictures and distractors (for other studies with the same rationale see Costa et al., 2009; Dell’Acqua et al., 2010). Interestingly, the effect resembles an N400 effect, even though it is much shorter than a typical N400 effect. Therefore, the question arises whether the ERP effect around 290 ms could also be a pure semantic facilitation effect caused by semantic priming of the related distractor. However, semantic priming and semantic relatedness has been found to lead to reduced negative activation, both in visual word processing and word production studies (e.g., Bentin et al., 1985; Holcomb and Neville, 1990; Chauncey et al., 2009; Blackford et al., 2012). The enhanced negativity of the related distractors compared to the unrelated distractors in our study is not compatible with this suggestion, because the effect is in the opposite direction to the semantic priming effect. It is therefore unlikely that our effect around 290 ms is a semantic priming instead of a lexical retrieval effect.

The second ERP effect (440–510 ms) falls into the window of phonological code retrieval and syllabification. The final two ERP effects (520–560 ms and 630–670 ms post stimulus onset) fall into the latter time window as well, but also fit the time window of self-monitoring (but not of fully prepared phonological responses). These later effects could also be part of a domain-general control mechanism outside language processing, which has been argued to be part of the PWI paradigm (Piai et al., 2013; Janssen et al., 2015). This is especially the case for the two anterior effects around 470 ms and 660 ms.

For the interpretation of our oscillatory power changes, it is worth discussing what theta and beta power increases have been argued to reflect. As noted in the introduction, an increase in frontal theta power has been related to increased frontal control mechanisms (e.g., Cavanagh and Frank, 2014). The increase in frontal theta power (450–540 ms) falls into the window of phonological code retrieval and syllabification. This increase might therefore reflect the suppression of phonologically encoding the related distractor or the effort of not mixing the phonological codes of picture and distractor.

Beta activity is well known to be decreased in preparation to movements, for instance, limb movements (e.g., Hari and Salmelin, 1997). It has also been found for the preparation of speech production (e.g., Salmelin and Sams, 2002; Saarinen et al., 2006; Gehrig et al., 2012; Jenson et al., 2014). Beta power decrease in these tasks is understood to reflect the preparation of the motor response and the readiness to move. While beta band synchrony has been related to a state of maintenance of posture or status quo (e.g., Engel and Fries, 2010), asynchrony has been related to readiness to move (Gilbertson et al., 2005; Tzagarakis et al., 2010). Response uncertainty in the case of competing potential responses is associated with less reduction of beta power (Tzagarakis et al., 2010). Beta band activity has also been related to cognitive processes. Engel and Fries (2010) have suggested that the active maintenance of a cognitive set and endogenously driven top-down attention processes, for instance in case of ambiguous stimuli, are associated with increase in beta band activity. Similarly, in a review of beta frequency effects in language processing, Weiss and Müller (2012) pointed out that beta power decreases can be related to maintenance of cognitive processes, top-down attention (e.g., attention to more important words) as well as semantic binding during lexical-semantic retrieval processes (for the latter, see, e.g., Luo et al., 2010).

These observations suggest that higher beta power in the related condition versus the unrelated condition in our study might reflect higher response uncertainty, endogenously driven top-down attention processes due to relatedness of the distractor and the maintenance of two active responses, and/or semantic binding during lexical-semantic retrieval processes. Interestingly, beta band effects occurred at various times during response preparation, from at least the timing of lexical retrieval (around −550 ms pre-speech onset) right before phonetic encoding (up to −155 ms pre speech onset). This suggests that response uncertainty is maintained until phonetic encoding. The earliest beta power difference occurred between 40 and 110 ms and falls into the time window of visual and semantic processing. This effect seems too early to reflect attention or state maintenance processes, because at this early stage words and pictures are still being recognized. But it cannot fully be ruled out that the processing system is able to adjust attention and maintenance processes with this rudimentary information. Dell’Acqua et al. (2010) suggested for a very early ERP effect that it might reflect feedback processes from the semantics of the picture to the processing of the distractor word. This fits with previous findings that beta decreases have been found for semantic binding and neural binding across domains (here: visual words, pictures and semantics) (see Weiss and Müller, 2012).

How do these results fit with the two accounts of the PWI? In line with the lexical competition account, we found both an ERP and time frequency effect during the time window of lemma retrieval. But given the additional effects, the story seems more complex. A response to the distractor does not seem to be fully suppressed after lemma retrieval. This suggests that the related distractor does not only increase lexical competition during lemma access. Later effects fit with the suggestion by Starreveld and La Heij (1996) that the PWI is related to the selection of the phonological word-form representation and potentially affects self-monitoring processes. While the lexical competition account does not predict our later effects, it needs to be pointed out that ‘late’ effects of semantic interference can be accounted for in some models of word production that assume lexical competition, like the model by Levelt et al. (1999).

The response exclusion account assumes two effects, i.e., semantic priming of the picture by the related distractor and a late effect when a fully planned response to the distractor needs to be excluded (Finkbeiner and Caramazza, 2006; Mahon et al., 2007; Janssen et al., 2008). As mentioned above, our very early finding of a decrease in high beta power (40–110 ms post stimulus onset) might potentially reflect such semantic ‘priming’ effect. But, as explained above, the ERP effect around 290 ms is very unlikely a semantic (priming) effect because it is in the opposite direction of a semantic priming effect. In contrast, the increase of high beta band activity between −175 and −155 ms before speech onset and the interpretation of increased beta band power to reflect response uncertainty fits the idea of a response exclusion mechanism of fully formed phonological words for both the picture and the distractor. However, our earlier effects suggest that the response exclusion explanation is not sufficient.

Taken together, our results suggest that semantic distractors affect the processing of the target pictures in more than one way and that both accounts of semantic interference, i.e., lexical competition and response exclusion, seem to capture a part, but not all aspects of the semantic interference effect. This conclusion is in accordance with findings using fMRI (de Zubicaray et al., 2012a,b) and also with the finding that superimposed distractors affect picture naming at various points during response preparation (Bürki, 2017).

Comparing our results with previous findings for semantic distractors in PWI paradigms, our ERP effect around 290 ms resembles previous results in terms of timing (Dell’Acqua et al., 2010; Aristei et al., 2011; Hoshino and Thierry, 2011; Zhu et al., 2015; Wong et al., 2017). However, the nature of the effect only matches that by Aristei et al. (2011), who report an increased bilateral anterior negativity for related distractors relative to unrelated distractors between 200 and 400 ms. In contrast, others found more negative activity for unrelated distractors relative to related distractors in similar time windows (e.g., Dell’Acqua et al., 2010; Hoshino and Thierry, 2011; Zhu et al., 2015; Wong et al., 2017). It is unclear how exactly the differences in the results of the studies arose. Given that our effect was not affected much by our artifact cleaning procedure (Porcaro et al., 2015), speech artifacts do not seem to be responsible for the difference. But procedural differences between the studies might be the cause. For instance, while Dell’Acqua et al. (2010) and Hoshino and Thierry (2011) left the stimuli on the screen, our stimuli disappeared after 250 ms. Aristei et al. (2011) presented distractors auditorily and −150 ms before the pictures, meaning early access, but no opportunity for re-processing of distractors. This is similar to removing distractors early and might explain similarities of their results with ours. However, Aristei et al. (2011) study is also the only study that presented their pictures in semantically homogeneous and heterogeneous blocks. Furthermore, Hoshino and Thierry (2011) study involved bilingual instead of monolingual participants. Such methodological differences might change the way pictures and words interact with each other and/or which words are entering the competition process. Future research will need to investigate whether methodological differences can explain the different ERP results, especially whether keeping the stimuli on the screen until response has an effect on EEG effects.

In terms of oscillatory activity, there are very limited previous results. The short increase in frontal theta power has not been reported before (but see the long-lasting increase in theta power in Shitova et al., 2017, who compared picture of identical with related distractors words). The beta band difference of Piai et al. (2012) for categorically related versus unrelated distractors between 230 and 370 ms resembles one of our effects, both in terms of nature and timing (275–340 ms). Piai et al. (2012) argued that the effect is in line with the lexical competition account due to its timing. While we agree that this effect is likely related to lexical competition, it is not the only effect in our study, which means it does not tell the whole story.

Overall, our effects seem to be relatively short and small compared to previous findings and compared to our strong behavioral effect. In addition, we found more effects than previous studies. It is possible that this is due to the shortened timing of our stimulus presentation. It is also interesting to see that ERP and time frequency analyses showed effects in partly different time periods, meaning that they seem to reflect different aspects of the underlying processes. Most importantly for the present study, we found an effect close to response onset only in the time frequency domain, not in ERPs. The study of oscillatory activity next to ERPs therefore seems to be a fruitful combination that should be explored more often.

We had previously shown that the SAR-ICA procedure for the removal of speech artifacts (Porcaro et al., 2015) that we applied in this study was very successful in removing speech motor related artifacts from the EEG. It is not certain whether other methods might lead to different results, especially in response-locked analyses. For instance, Ouyang et al. (2016) presented an alternative approach, using residue iteration decomposition. They argue that their method was superior to an ICA-based method. But even if our method might not have removed all speech motor artifacts from the EEG signal, it is rather unlikely that our effects, including the one close to speech onset, were due to speech motor artifacts. This is because all participants named the same pictures in the related and unrelated conditions and it is unlikely that the procedure would attenuate artifacts better in one than in the other condition.

## Conclusion

To conclude, cleaning our data from speech motor artifacts allowed to investigate later processing stages in the PWI paradigm and therefore to address the nature of the semantic distractor effect. In particular, we were able to investigate processes close to speech onset, which are important for the response exclusion account. Our results suggest that the semantic distractor effect is actually a combination of various effects. Lexical competition accounts and the response exclusion account capture different parts, but not all aspects of the behavioral effect. Having said this, our results generally support the traditional conclusion that the PWI paradigm involves competition between categorically related lexical representations and cannot be reduced to later processes such as monitoring processes or a response exclusion mechanism. The results are therefore in line with the idea of lexical selection by competition.

## Data Availability

The datasets generated for this study are available on request to the corresponding author.

## Author Contributions

AK designed the study. AK and CP contributed equally to its analysis and write-up. MTM contributed to the analysis of the data.

## Conflict of Interest Statement

The authors declare that the research was conducted in the absence of any commercial or financial relationships that could be construed as a potential conflict of interest.

## Footnotes

1 https://openwetware.org/wiki/Mass_Univariate_ERP_Toolbox

2 The same analysis applied before cleaning the EEG from speech motor artifacts (Raw Data only, corrected for eye movements) led to similar results around 290 and 660 ms, but did not lead to any significant differences at 470 and 540 ms.

## Appendix A: Experimental material

**Table:**

Target	Semantically Related distractor	Semantically Unrelated distractor
arrow	spear	radish
banana	orange	spear
bear	tiger	duster
bone	muscle	table
broom	duster	orange
brush	mirror	muscle
cheese	butter	tiger
comb	shampoo	baker
cook	baker	wallpaper
curtain	wallpaper	butter
dress	shirt	statue
flag	banner	shampoo
fork	napkin	mirror
fountain	statue	shirt
foot	ear	banner
couch	table	napkin
onion	radish	piano
organ	piano	jumper
plane	helicopter	hamster
rabbit	hamster	helicopter
snail	worm	trumpet
tent	shelter	ear
trousers	Jumper	worm
violin	Trumpet	shelter
