# Supplementary material for: Early and Late Effects of Semantic Distractors on Electroencephalographic Responses During Overt Picture Naming
Source: Front Psychol. 2019 Mar 28;10:696. doi: 10.3389/fpsyg.2019.00696 (PMC6447652; doi:10.3389/fpsyg.2019.00696)
Supplement: Supplementary file 1 [file Table_1.DOCX]

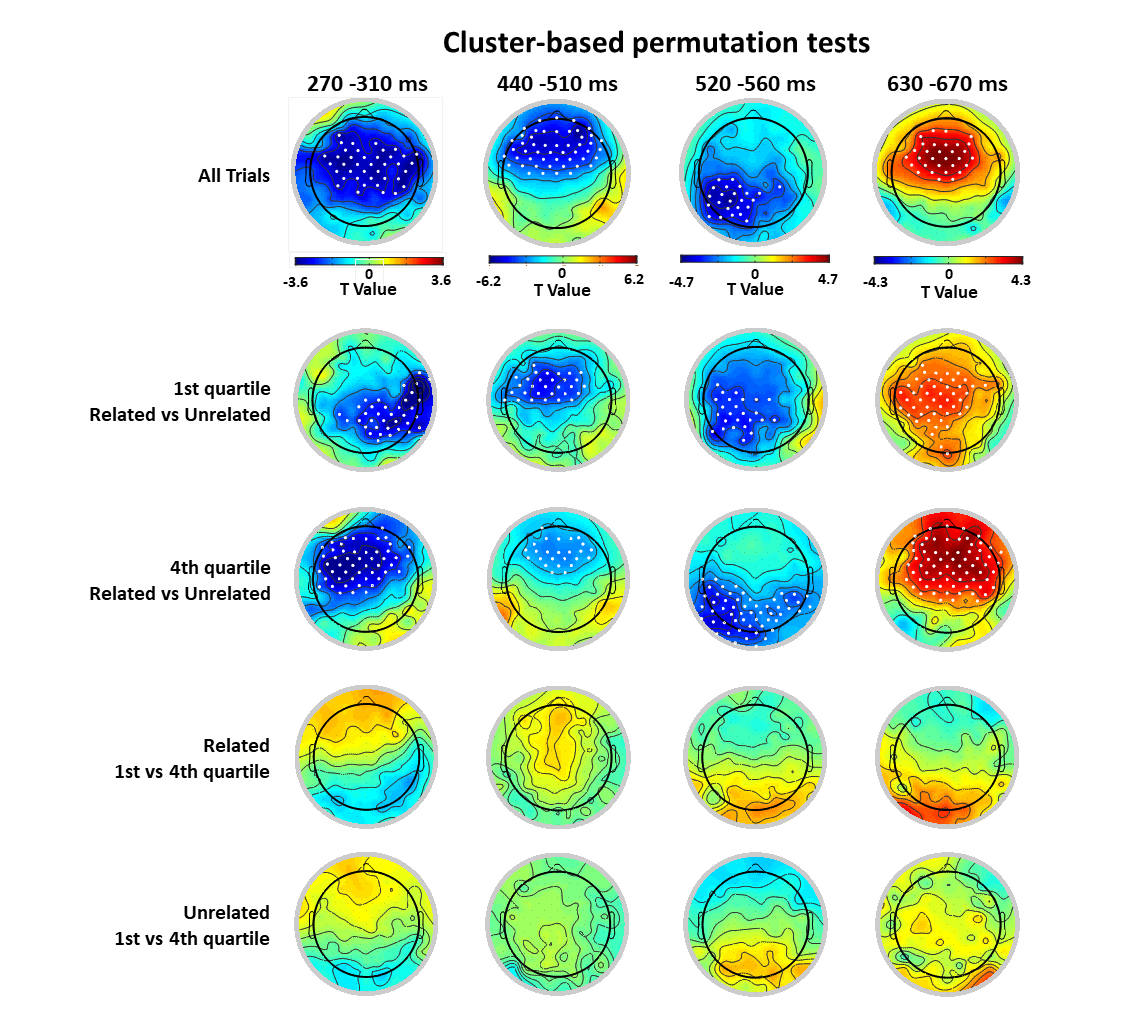


Figure S1. Cluster-based permutation tests of ERP differences between Semantically Related and Unrelated distractor conditions for the 1^st^ and 4^th^ quartile of the trials.

The upper row of panels shows the scalp distributions of the stimulus-locked ERP effects (270 – 310 ms, 440-510 ms, 520 – 560 ms, 630 – 670 ms) comparing related and unrelated semantic distractors and for all trials combined (see also Figure 2). The second and third row of panels show the scalp distribution of the effects for only the 1^st^ and 4^th^ quartile of the trials. The fourth and fifth row of panels show the absence of significant differences between the 1^st^ and 4^th^ quartile of the trials, for related and unrelated distractors separately. White dots indicate electrodes with significant differences determined by two-tailed cluster mass permutation tests with a family-wise alpha level of 0.01 (<https://openwetware.org/wiki/Mass> Univariate ERP Toolbox).
