# Supplementary material for: Early and Late Effects of Semantic Distractors on Electroencephalographic Responses During Overt Picture Naming
Source: Front Psychol. 2019 Mar 28;10:696. doi: 10.3389/fpsyg.2019.00696 (PMC6447652; doi:10.3389/fpsyg.2019.00696)
Supplement: Supplementary file 2 [file Table_2.DOCX]

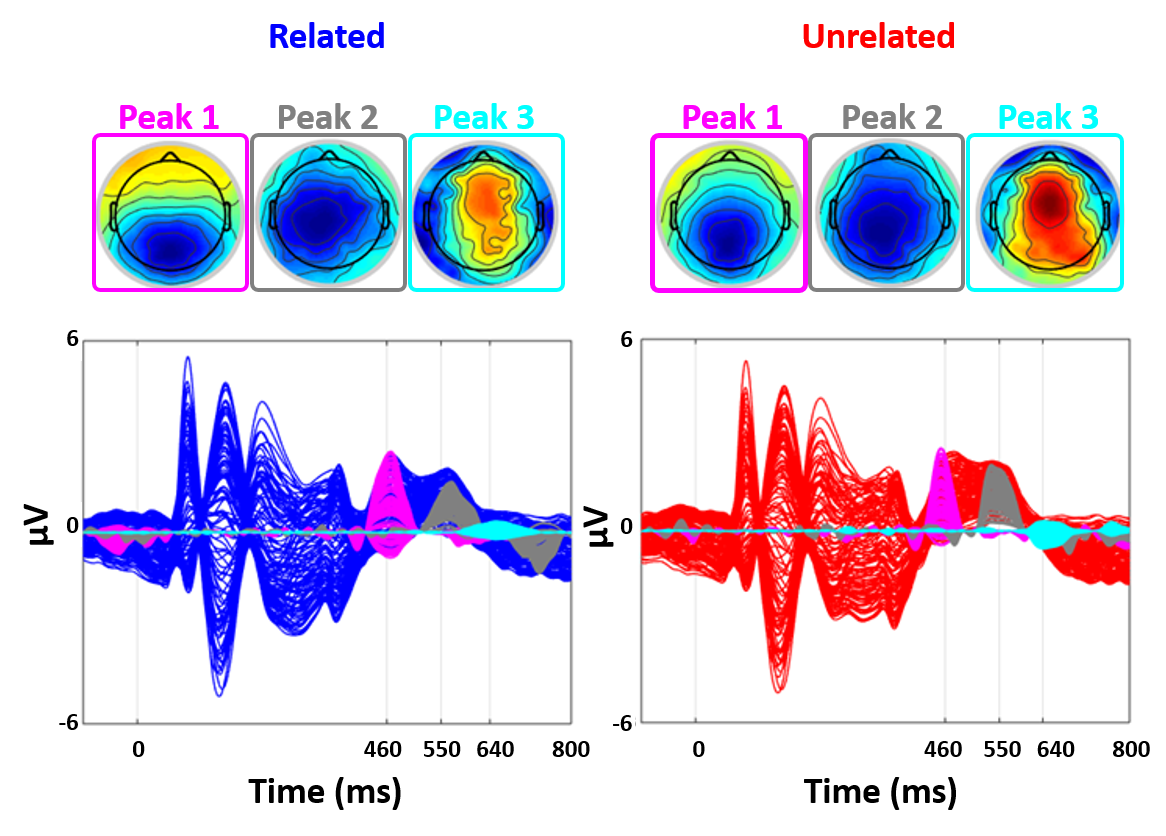


Figure S2. Independent components (ICs) underlying the peaks of the three late ERP effects of distractor relatedness. The upper row of panels shows the scalp distributions of the ICs underlying peaks at 460, 550, and 640 ms respectively. The lower panel shows the butterfly representation of all 128 electrodes for the ERPs to related (blue) and unrelated (red) distractors and for the ICs underlying peak 1 at 460 ms (magenta), peak 2 at 550 ms (grey) and peak 3 at 640 ms (cyan).
